# Quality of life in children and adolescents in Central Kenya: associations with emotional and behavioral problems

**DOI:** 10.1007/s11136-019-02099-8

**Published:** 2019-01-17

**Authors:** Dorcas N. Magai, Hans M. Koot

**Affiliations:** Department of Clinical, Neuro- and Developmental Psychology, Vrije Universiteit Amsterdam, Van der Boechorststraat 1, 1081 BT Amsterdam, The Netherlands

**Keywords:** Quality of life, Emotional and behavioral problems, Child, Adolescent, Kenya

## Abstract

**Purpose:**

To assess the quality of life (QoL) of children and adolescents in Kenya as rated by parents and youth themselves, and examine how QoL is related to emotional and behavioral problems (EBP).

**Method:**

QoL and EBP reports were obtained from 1022 Kenyan parents and 533 adolescents living in the country’s Central Province. Parents with children between 6 and 18 years completed the Pediatric Quality of Life Inventory (PedsQL) and the Child Behavior Checklist, while the adolescents (12–18) completed the PedsQL and the Youth Self-Report.

**Results:**

Parent-reported QoL in Kenyan youth was somewhat above that of US standardization samples, while levels of adolescent self-reports were well within the range of those from most high- and middle-income countries. Average adolescent girls’ self-reports were lower on all QoL scales than boys. QoL in children/adolescents with clinical to borderline levels of EBP (cf. multicultural norms, Achenbach and Rescorla, 2007) was lower than QoL in agemates with normal levels of EBP. Regression analyses indicated unique associations of QoL with parent-reported withdrawn/depressed, somatic complaints, attention problems, and aggressive behavior, and with adolescent self-reported somatic complaints, attention problems, and rule-breaking behavior.

**Conclusion:**

QoL levels were well within ranges of other countries. Moreover, associations of QoL with EBP indicated that those with borderline/clinical levels of EBP had a much lower QoL most notable for those with somatic complaints and attention problems. Mental health providers might focus on interventions that reduce EBP in Kenyan children and adolescents and simultaneously reduce the risk of lowered QoL.

## Introduction

Quality of life (QoL) has been described as both a subjective evaluation of an individual’s well-being as well as objective descriptions of individuals and their circumstances that are associated with subjective well-being and may be amenable to change through service and policy [1, 2]. Child and adolescent QoL comprises subjective dimensions such as experience of psychological, physical, and social functioning; and objective conditions such as living, employment, and educational facilities [3, 4]. In this paper, we focus on QoL as the subjective experience of physical, emotional, and social functioning.

QoL is important to examine in youth not just because it reflects well-being broadly, but also because it conveys information about their essential daily functioning [4]. Physical QoL addresses how youth are able to engage in daily activities, whereas psychosocial QoL captures how they feel about and perceive themselves and their lives, the quality of their relationships, and how well they function in critical roles, such as a student [5]. Although some population studies on adults’ QoL have been performed in African countries [6–8], and multiple on high-risk groups, little is known on QoL in African children and adolescents from the general population. To fill this gap, this study addressed QoL of children and adolescents in Kenya as rated by parents and youth themselves.

Many Kenyan children and adolescents have been exposed to different traumatic events given the social tensions and violence that the country has experienced [9]. Moreover, the children and adolescents are faced with high rates of school drop-out, children wandering on the streets, high rates of crimes especially in the youth population, and high unemployment rates [10, 11]. Given that conditions like these are all strongly associated with a country’s economic status, and that well-being is clearly related to income both within and across countries [12], QoL in Kenyan youths might be expected to be reduced, when compared to those living in high- or middle-income countries like the USA or China, respectively, but similar to that in other low-income countries like India. For example, while the 2017 median US annual family income was USD 59,000 [13], more than half of Kenyan families have an annual income less than USD 1200 [14]. As demonstrated recently [15–17], income disparities and their associated conditions may seriously affect children’s QoL.

Psychopathology is one of the conditions that has shown to be an important correlate of QoL in children and adolescents. Although little is known about the QoL in children and adolescents with emotional and behavioral problems (EBP) in the general population, a recent review [18] demonstrated that QoL in children and adolescents with psychological and psychiatric problems do have a seriously reduced QoL. An Australian study [19] reported that children with major depressive disorder, conduct disorder, and attention deficit disorder (ADHD) had worse QoL in several domains compared to children without mental illness. Similarly, a Dutch study [20] found that children with psychopathology had significantly lower functioning on different QoL domains compared to a healthy sample. These studies, therefore, indicate that children and adolescents with psychopathology not only have more impaired QoL than their peers from the general population but also have their QoL comparable to or even poorer than pediatric patients with physical conditions.

Despite the general knowledge that children and adolescents with psychopathology have lower QoL and lower levels of functioning compared to those without, this finding is mainly based on studies that have been conducted in developed nations, and little is known about this association in African countries. One study performed in Nigeria found small but significant associations between QoL and general mental health problems in an adolescent school sample [21]. A recent study [22] demonstrated that EBP in Kenyan children and adolescents are highly prevalent compared to multicultural standards for parent reports, with 27% and 17% scoring in the borderline and clinical range, respectively. In the present study, we aimed to examine how the QoL of Kenyan children and adolescents is related to their EBP. Addressing this question may be informative about the meaning of parent- and youth self-reports on QoL, as elevated levels of EBP are expected to reduce overall QoL.

To achieve these aims, the study included children and adolescents ages 6–18 years from a large random epidemiological sample, and used standardized parent-proxy and youth self-reports on both QoL and EBP. All analyses accounted for age and gender differences, as applicable.

## Method

### Participants

The study was part of the Kenya Child and Adolescent Mental Health Study and included parents and adolescents living in the Central Kenya Kiambu and Nyeri Counties. Within these counties, random sampling was set up targeting an equal distribution of children across age (6–18 years) and gender, and power calculations were based on the study’s prime focus on population prevalence of child/adolescent psychopathology. Of the 1068 parents and 576 adolescents selected, 46 adolescents (8.0% of target sample) and their parents (4.3% of target sample) could not be reached as either the children were in boarding schools or their parents were away during the interview. Half of the children on whom information was obtained in this study (50.4%) were girls. Of the 1022 children and adolescents included, 47.8% were between 6 and 11 years, while 52.2% were between 12 and 18 years. Further information on sampling and demographics is given in Magai et al. [22].

## Measures

### The Pediatric Quality of Life (PedsQL) inventory

The PedsQL is a 23-item questionnaire for measuring QoL in children and adolescents between ages 2 and 18 [5, 23], with fully parallel, age appropriate versions for parent report and youth self-report. The PedsQL Generic Core Scales (those for ages 5–7, 8–12, and 13–18 for parent report, and for ages 13–18 for youth self-report) were used in this study to assess the occurrence of problems in different domains of functioning including the physical, emotional, social, school, and overall psychosocial functioning. Each item was answered on a 5-point scale ranging from 0 (never) to 4 (almost always). Items were reverse-scored and transformed to 0–100 scale (0 = 100, 1 = 75, 2 = 50, 3 = 25, 4 = 0), in a manner that higher scores indicate better functioning based on the item scores. The parent version was completed by one of the parents of all children (aged 6–18) in the sample, while the youth version of the instrument was completed by adolescents aged 12–18 years. Good reliability and validity have been reported for the PedsQL subscales and total score (Cronbach’s alpha = 0.72–0.89 for adolescents and 0.76–0.92 for parents; [5]). The factorial invariance of across ethnic groups has been demonstrated as well [23]. In this study, acceptable to good Cronbach’s alphas were found (mean 0.72; range 0.58–0.85 for adolescents and mean 0.72; range 0.55–0.84 for parents) for the PedsQL subscales and total score (see Tables 1, 2).

**Table 1:** Mean (SD) parent-reported PedsQL scale scores by gender and age groups

	Total group	Boys	Girls	Boys	Girls	Gender × age	*p*	Contrast
		6–11	6–11	12–18	12–18	*F*		
*N*	1020	243	245	263	269			
Physical (0.76)	91.6 (15.3)	91.4 (15.4)	90.9 (15.1)	92.9 (14.8)	91.3 (15.6)	0.85	0.467	Na
Emotional (0.75)	87.3 (18.8)	85.4 (20.0)	87.6 (18.7)	90.5 (16.2)	85.5 (19.9)	4.21	0.006	3 > 1, 2
Social (0.55)	86.8 (18.8)	84.2 (20.2)	84.4 (19.4)	89.7 (17.5)	88.5 (17.6)	5.87	0.001	3 > 1, 2 4 > 2
School (0.66)	88.7 (18.6)	87.0 (20.6)	88.9 (16.7)	90.2 (17.4)	88.5 (19.3)	1.25	0.289	Na
Psychosocial (0.76)	87.5 (15.2)	85.5 (16.3)	87.0 (15.0)	90.1 (13.6)	87.3 (15.4)	4.25	0.005	3 > 1
Total (0.84)	88.9 (13.6)	87.4 (14.5)	88.3 (13.4)	91.1 (12.3)	88.7 (13.8)	3.42	0.017	3 > 1

Scale alphas and SD in brackets. Gender × age groups are denoted 1–4, from left to right. Contrasts cf. Tukey post hoc test (*p* < 0.05)

*Na* not applicable

**Table 2:** Mean (SD) youth-reported PedsQL scale scores by gender

	Total group	Boys	Girls	*F*	*p*
*N*	524	261	263		
Physical (0.74)	85.8 (18.8)	86.9 (19.4)	84.6 (18.1)	1.88	0.171
Emotional (0.74)	82.1 (21.6)	85.2 (20.7)	79.0 (22.0)	11.07	0.001
Social (0.58)	81.4 (21.5)	83.7 (21.0)	79.1 (21.9)	5.74	0.017
School (0.63)	87.6 (18.8)	88.9 (18.5)	86.3 (19.0)	2.46	0.118
Psychosocial (0.76)	83.5 (16.8)	85.8 (16.5)	81.2 (16.8)	10.18	0.002
Total (0.85)	84.2 (15.7)	86.3 (15.8)	82.3 (15.3)	8.50	0.004

Scale alphas and SD in brackets

### Child Behavior Checklist/4–18 (CBCL) and Youth Self-Report (YSR)

The CBCL [24, 25] was used to obtain standardized parental reports of children’s and adolescents’ problem behaviors. The problem section of both scales consists of 118 3-point items describing behavioral and emotional problems, and takes 25–30 min to complete. Parental caregivers were asked to rate the occurrence of their children’s problem behaviors in the past 6 months by circling 0 (for not true), 1 (for somewhat or sometimes true), or 2 (for very true or often true). For each age- and gender group, the current study used existing 8 syndromes to represent syndromes of problems that tend to occur together as identified in other studies [26]. The CBCL has worldwide established good psychometric properties (see http://www.aseba.org), and its factorial structure and psychometric properties have been replicated for the Kenyan population of children and adolescents [22].

The YSR [25, 27] YSR has the same structure as the CBCL/6–18 and is used to obtain responses from the youth about their EBP. The YSR problem scale has 103 items which the youth respond to by circling 0 if the statement is not true, 1 if the statement is somewhat or sometimes true, and 2 if the statement is very true or often true to them. The YSR has the same 8 syndrome scales as the CBCL, and takes 25–30 min to complete. The YSR has been found reliable to use as indicated by the international generalizability of its structure of youth EBP and has good psychometric properties (see http://www.aseba.org). Factorial structure and psychometric findings were replicated for the Kenyan population of children and adolescents [22]. For information on item content of CBCL and YSR, see http://www.aseba.org/forms.html.

To identify those with scores in the borderline or clinical range of the CBCL and YSR, we used the cut-off scores based on multicultural norms for both instruments described by Achenbach and Rescorla [28].

### Procedures for data collection

The research proposal was approved by the scientific and ethical review committee of the Faculty of Behavioral and Movement Sciences of Vrije Universiteit Amsterdam. Further approval was received from the National Commission for Science, Technology and Innovation (NACOSTI) in Kenya, which also approved the ethical standards of the study and issued the needed research permit. Data collection was performed by four trained research assistants supervised by the first researcher. After establishing eligibility of the participants, oral and written informed consent was obtained. Parents (mothers 88.3%, stepmothers 0.6%, grandmothers 6.0%, aunts 1.5%, other 1.8%) and youths completed the questionnaires independently, and questions were fully answered in accordance with the instruments’ manual. For those who appeared to have difficulty reading and understanding the written Swahili text, items were read aloud by the research assistant, who also filled out the forms assuming that difficulties in reading also imply difficulties in writing. For a full description of the procedures for data collection in this study, we refer to Magai et al. [22].

### Statistical analysis

First, descriptive statistics including means, standard deviations, and percentages were computed to examine the demographic characteristics of the participants.

Secondly, using the instructions of the PedsQL and CBCL/YSR manuals, raw scores for the instruments’ scales were computed by summing the ratings for the items in each scale. To assess the internal consistency of the PedsQL, Cronbach’s α was computed for each version’s scales.

To assess the effect of demographic variables (age groups 6–11 and 12–18, and gender) on QoL reported by parents and adolescents, analyses of variance (ANOVA) with gender and age group as factors were conducted on each PedsQL scale.

To address the question to what extent CBCL and YSR problem scores are associated with a lowered QoL in Kenyan children and adolescents, first, we computed PedsQL scores for those scoring below and above the multicultural borderline/clinical cut-points of the CBCL and YSR [28], respectively. Multivariate analysis of variance (with age and gender) was then used to examine to what extent CBCL and YSR problem scores are associated with a lowered QoL in Kenyan children and adolescents. To assess the association of CBCL and YSR Internalizing Problems, Externalizing problems, and Total Problems with overall parent- and youth-reported QoL, Pearson correlations were computed. Lastly, to address the question which CBCL and YSR problem types specifically affected QoL most, we regressed PedsQL total scores for parent and youth informants on CBCL and YSR subscale scores, respectively, controlling for age and gender.

## Results

### Parent- and youth-reported PedsQL scores by age and gender

Means and standard deviations (SD) of parent-reported PedsQL scores are presented in Table 1. Significant group (ages 6–11, 12–18; gender) effects were found for all but the physical and school scales. Post hoc contrasts indicated significantly higher scores for boys 12–18 as compared to both younger groups on emotional and social functioning, and as compared to younger boys on psychosocial and total functioning. Moreover, older girls were scored higher than younger girls on social functioning.

Mean youth-reported PedsQL scale scores and standard deviations are presented in Table 2. ANOVA indicated that the effect of gender was significant on four scales, i.e., emotional, social, psychosocial, and total, all indicating lower scores in girls than in boys.

Pearson correlations between child age and youth-reported PedsQL scale scores indicated a weak, positive association between physical and age (*r* = 0.12, *p* = 0.006). Also, paired t tests between parent-reported and youth-reported scales indicated low and non-significant correlations between informants (*r*s ranging from 0.01 to 0.06) and significant differences in means for all but the school scale (*t* values ranging from 4.76 to 6.30, all *p* = 0.000). Therefore, based on the findings for parent- and youth reports and their comparison, further analyses with these data controlled for effects of gender and age, and analyses were performed separately for PedsQL parent reports and youth reports.

### PedsQL scale scores by borderline/clinical CBCL and YSR scores

Multivariate analysis of variance (with age and gender as covariates) showed significant effects of borderline/clinical status on both parent-reported (Pilais Trace, *F* = 34.80, *p* < 0.000) and youth-reported (Pilais Trace, *F* = 14.72, *p* < 0.000) QoL. These results indicate that elevated scores on both parent- and youth-reported behavioral and emotional problems are strongly associated with lowered QoL reported by the same informant. Univariate analyses were significant for all scales (*p* < 0.000) and indicated small to large effects, ranging from 0.03 for physical to 0.16 for psychosocial for parent reports, and ranging from 0.06 for social to 0.14 for total functioning for youth reports (Table 3). Thus, also in Kenyan children and adolescents CBCL and YSR problem scores in a range that distinguish best between children referred vs. non-referred for mental health services [29] are moderately to strongly associated with lowered personal and societal functioning as indicated by the PedsQL.

**Table 3:** Mean (SD) PedsQL scale scores by borderline/clinical CBCL and YSR total problem scores (BCTPS)

	Below BCTPS	Above BCTPS	F*	ES
N CBCL	743	275		
N YSR	430	76		
Physical				
C	93.4 (13.7)	86.9 (17.9)	34.8	0.03
Y	88.3 (16.7)	73.7 (21.3)	44.0	0.08
Emotional				
C	91.4 (15.5)	75.9 (22.1)	151.0	0.13
Y	84.5 (20.6)	68.6 (21.6)	38.0	0.07
Social				
C	89.8 (16.8)	79.2 (20.9)	63.3	0.06
Y	83.9 (19.7)	69.0 (24.6)	33.3	0.06
School				
C	92.7 (15.4)	78.2 (21.3)	140.0	0.12
Y	90.7 (15.7)	73.1 (23.0)	68.6	0.12
Psychosocial				
C	91.2 (12.5)	77.5 (16.9)	191.1	0.16
Y	86.2 (14.8)	70.1 (16.7)	74.0	0.13
Total				
C	92.0 (11.4)	80.7 (15.0)	159.1	0.13
Y	87.0 (13.4)	71.2 (15.8)	83.3	0.14

SD in brackets

*BCTPS* scores below and above borderline/clinical cut-points of CBCL and YSR Total Problem Scale based on multicultural norms (cf. [28]); *C* CBCL Total Problems Borderline Score, *Y* YSR Total Problems Borderline Score, *ES* effect size

*All *p* < 0.000

Pearson correlations computed to test for the associations between CBCL and YSR Internalizing Problems, Externalizing problems, and Total Problems with overall parent- and youth-reported yielded significant (*p* < 0.000) medium-sized correlations of *r* = − 0.38, − 0.38, and − 0.41, for CBCL Internalizing, Externalizing, and Total Problem scores, and of *r* = − 0.42, − 0.42, and − 0.44 for their respective YSR counterparts, indicating that in Kenyan children/adolescents both EBP add to limitations in overall functioning as assessed by parents and youths themselves. Pearson correlations of the PedsQL total score with CBCL and YSR subscales ranged from 0.30 to 0.40 for parent reports, and from 0.31 to 0.40 for youth self-reports.

Regression analyses to identify the CBCL and YSR problem scales that affected QoL while controlling for age and gender demonstrated that independent of all other CBCL problem scales, withdrawn-depressed, somatic complaints, attention problems, and aggressive behavior were associated with parent-rated overall QoL (*p* ≤ 0.05). Together, these variables explained 20% of the variance, while YSR problem scales somatic complaints and rule breaking were independently associated with overall youth-rated QoL (*p* < 0.05), explaining 22% of its variance (see Table 4). This indicates that also for Kenyan children and adolescents CBCL and YSR scales from the whole breadth of the problem spectrum are associated with limitations in functioning indicating lowered QoL.

**Table 4:** Regression of parent and youth-reported PedsQL total scores on CBCL and YSR scale scores

	B (SE)	Beta	*t* value	*p*
CBCL/YSR scale				
Anxious-depressed				
C	0.19 (0.14)	0.05	1.06	0.311
Y	− 0.28 (0.28)	− 0.06	1.01	0.292
Withdrawn-depressed				
C	− 0.45 (0.23)	− 0.08	− 1.97	0.050
Y	− 0.41 (0.34)	− 0.06	− 1.21	0.228
Somatic complaints				
C	− 0.55 (0.16)	− 0.13	− 3.47	0.001
Y	− 0.69 (0.29)	− 0.13	− 2.41	0.016
Social problems				
C	0.30 (0.19)	0.07	1.58	0.115
Y	0.43 (0.32)	0.08	1.32	0.187
Thought problems				
C	− 0.18 (0.19)	− 0.05	− 0.92	0.358
Y	− 0.47 (0.33)	− 0.09	− 1.44	0.151
Attention problems				
C	− 0.94 (0.17)	− 0.22	− 5.39	0.000
Y	− 0.66 (0.31)	− 0.10	− 1.82	0.070
Rule breaking				
C	− 0.96 (0.19)	− 0.02	− 0.50	0.617
Y	− 0.75 (0.31)	− 0.14	− 2.42	0.016
Aggressive behavior				
C	− 0.42 (0.14)	− 0.14	− 2.88	0.004
Y	− 0.41 (0.28)	− 0.09	− 1.48	0.140

Entries are obtained from multivariate regression analyses within each informant

*C* CBCL Scale Score, *Y* YSR Scale Score

The same analyses were performed with the parent- and youth-reported PedsQL psychosocial summary score to check whether somatic complaints entered the equation as the PedsQL total score also includes physical functioning items. These analyses demonstrated that all significant associations found in the analyses with the overall QoL held, and that for the parent PedsQL and CBCL, social problems entered the equation (*β* = 0.12, *t* = 2.59, *p* = 0.01). Additionally, for the youth PedsQL, YSR attention problems came in (*β* = − 0.13, *t* = − 2.42, *p* = 0.02). This indicates that across both informants somatic complaints are indeed associated with overall functional problems, independent of other types of EBP.

## Discussion

This is one of the first studies in a well-defined general population sample of African children and adolescents aged 6–18, using standardized developmentally appropriate measures of both QoL and psychopathology, both self- and parent-proxy reported and addressing age and gender differences (cf. [23, 26]).

According to the parents’ reports, the overall QoL in Kenyan children and adolescents as found in this study is somewhat above (0.6 SD) that of large US standardization samples [30]. Averages of adolescent self-reports were well within the range of those from high-income countries (like the USA [31], see also Petersen et al. [32]), most middle-income countries like Brazil, China, Iran, and Thailand [33–36], similar to those of adolescents from Nigeria [21], but higher than those from samples in other low-income countries like India [37] and Tonga [32]. However, adolescent girls’ self-reports showed lower averages on all QoL scales than boys. Analyses showed that QoL in children/adolescents with clinical to borderline levels of EBP was lower than QoL in agemates with normal levels of EBP. This association held for both self- and parent reports, with effect sizes in the medium to large range (except the physical scale in parent reports which was small). While parent- and self-reported QoL was significantly associated with all EBP scales, regression analyses indicated unique associations for parent-reported withdrawn/depressed, somatic complaints, attention problems, and aggressive behavior, and also unique associations for adolescent self-reported somatic complaints, attention problems (marginally), and rule-breaking behavior.

It is remarkable that adolescents in this study rated their QoL levels similar to those in both high- and middle-income countries, although this was also true for levels reported in the other study on sub-Saharan youths [21]. Thus, even the strongly adverse conditions reported for Kenyan youth do not seem to grossly affect population mean levels of adolescent-reported QoL.

Overall, parent ratings differed somewhat across gender × age groups, and were lower for younger children. However, the differences were small. The finding that adolescent girls rated lower than boys might indicate an association between elevated internalizing problems in girls and poorer QoL. Kenyan girls reported higher levels of internalizing problems [22] compared to their Kenyan male counterparts and higher than their female counterparts in other multicultural societies.

As expected, QoL was strongly affected by the presence of EBP in the borderline to clinical range. This converges with findings by Bastiaansen et al. [3] who also reported that EBP strongly affect different domains of functioning in children and adolescents. In that study, QoL of children and adolescents referred for mental health services was scored about 1 SD below that of non-referred agemates by both parents and youths themselves. Similar results were obtained in the present study when comparing those scoring above the borderline or clinical range of EBP with those scoring below. This indicates that despite a possible well-being paradox and similar to other cultures, QoL assessments in Kenyan children and adolescents are sensitive to special negative conditions. Our results also showed that somatic complaints, attention problems, and behavior problems/rule breaking are uniquely related with QoL. This partly concurs with the findings from the review study by Jonsson et al. [18] indicating that QoL is most affected in children with attention problems (ADHD), and findings reported by Bastiaansen et al. [20] indicating that children with attention deficit disorder, disruptive behavior problems, and anxiety and mood disorders had lower functioning compared to children with other mental disorders.

A possible explanation for the strong effects of EBP on QoL is that EBP affect functioning of children and adolescents in several ways. For example, children with somatic complaints are more likely to be absent from school, thus affecting their school functioning. Children with internalizing problems are more likely to be rejected by their peers, thus affecting their social functioning domains. Therefore, also in Kenyan children and adolescents CBCL and YSR problem scores in a range that best distinguish between children referred vs. non-referred for mental health services are moderately to strongly associated with lowered personal and societal functioning as indicated by the PedsQL. The fact that significantly lower mean PedsQL scores were obtained for children and adolescents with elevated EBP is an evidence of criterion validity. Consequently, mental health practitioners in Kenya can use the PedsQL to obtain information on potential effects of psychopathology in the whole breadth of the problem spectrum on children and youth referred to their practice.

A special finding in this study relates to the low cross-informant agreement across adolescent and parent ratings of QoL. Although it is generally agreed that subjective reports of QoL are preferred [4], several findings in this study were consistent across both informants, while only self-report revealed differences between boys and girls QoL. So, adolescents and their parents may provide both valid, but complementary information.

### Strengths and limitations of the study

This study has several strengths. First, to our knowledge, this is the first general population-based study that has been conducted in Kenya to investigate the QoL in children and adolescents, and its association with EBP. Therefore, the current study does not only increase our scientific knowledge about the level of QoL in Kenyan boys and girls of different ages, but also about EBP as a potential factor of influence. Secondly, the study is based on sound methodology with random selection of study locations, a large sample size with a well-balanced distribution across age and gender, and the use of well-validated internationally comparable instruments (PedsQL, CBCL, and YSR), making the findings of this study possibly generalizable not only to Kenya but also to other African countries with similar cultural background as Kenya. Thirdly, our study takes into account both the adolescent child and their female caregiver’s reports. The study, therefore, captures not only the necessary subjective view of the adolescent on his/her QoL, but adds the parents’ view which may be of particular importance for children in the younger age range.

However, it also has several limitations. Firstly, the study was only conducted in two out of the 42 counties in Kenya. Secondly, although cross-ethnically [38] or cross-culturally [26] validated, the instruments used in this study did not consider the possible cultural interpretation and related need for adjustment of items to fit the Kenyan context. It should be acknowledged that both child and parent reports on QoL may be influenced by many personal and situational factors [4, 39] that were not addressed in this study. Subjective ratings, including the self- and proxy reports of both QoL and EBP used in this study, may be subject to many different, often unknown influences. However, they are universally accepted as a method to tap psychological constructs, including QoL and EBP. In this study, we did not control for any background variables as explanation of differences in QoL—except for sex and age—as it was not our aim to test any potential sources of bias. Additionally, father reports were not considered in this study, as fathers were generally not available as caregiver informants, a situation well known in sub-Saharan families [40]. Also, teacher reports of EBP were not included which may have reduced the view on QoL visible outside the home environment (e.g., schools).

### Study implications and conclusion

Our study findings suggest that subjective QoL is at well acceptable levels for most Kenyan children and youth but is strongly reduced in children and adolescents with EBP. Early identification, screening, and treatment of children with EBP is a task that should be given priority in Kenyan children to avoid the risk of developing future functional and more serious mental health problems. Particular attention is needed in regard to somatic complaints as well as emotional problems, attention problems, and disruptive behavior as these seem to affect QoL most. Since additional risk factors data and need for support assessment are also available in this study, our next task will be to examine how these affect QoL next to EBP.

In summary, this study showed that QoL levels as rated by parents were higher than those reported by parents in the USA, but lower for adolescent girl self-reports. Moreover, associations of QoL with EBP indicated that those with borderline/clinical levels of EBP had a much lower QoL most notable for those with somatic complaints and attention problems. Mental health providers might focus on interventions that reduce EBP in Kenyan children and adolescents and simultaneously reduce the risk of lowered QoL. Alternatively, they might focus on influencing child and family factors associated with better or improved QoL like child self-esteem or family support [39], even without improvement of psychopathology [41]. Additional studies are required to examine other factors associated with potential improvement of child and adolescent QoL in the Kenyan context.
